# Attojoule Superconducting Thermal Logic and Memories

**DOI:** 10.1021/acs.nanolett.4c06545

**Published:** 2025-03-10

**Authors:** Hui Wang, Niels Noordzij, Mischa Mikhailov, Stephan Steinhauer, Thomas Descamps, Eitan Oksenberg, Val Zwiller, Iman Esmaeil Zadeh

**Affiliations:** † Department of Imaging Physics, Delft University of Technology, 2628 CN Delft, The Netherlands; ‡ Single Quantum B.V., 2628 CH Delft, The Netherlands; § Department of Applied Physics, Royal Institute of Technology (KTH), SE-106 01 Stockholm, Sweden

**Keywords:** Superconducting device, logic gate, memory device, digital circuits

## Abstract

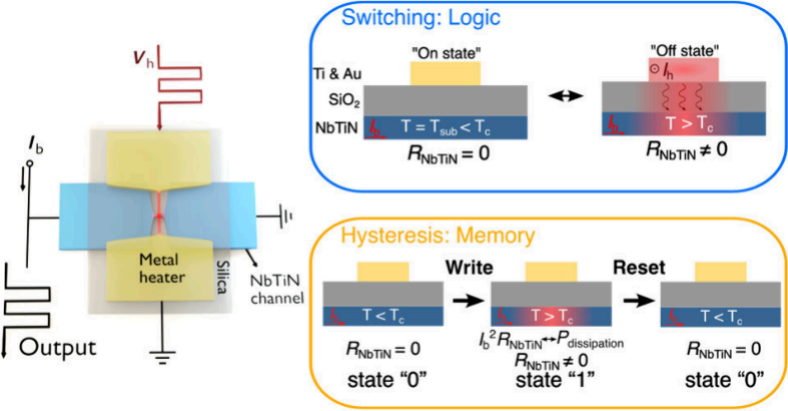

Due to stringent thermal budgets in cryogenic technologies such as superconducting quantum computers and sensors, electronic building blocks that simultaneously offer low energy consumption, fast switching, low error rates, a small footprint, and simple fabrication are pivotal for large-scale devices. Here, we demonstrate a superconducting switch with attojoule switching energy, high speed (pico-second rise/fall times), and high integration density (on the order of 10^–2^ μm^2^ per switch). It consists of a superconducting nanochannel and a metal heater separated by an insulating silica layer. We experimentally demonstrate digital gate operations utilizing these nanostructures, such as NOT, NAND, NOR, AND, and OR gates, with a few femtojoules of energy consumption and ultralow bit error rates <10^–8^. In addition, we build energy-efficient volatile memory elements with nanosecond operation speeds and a retention time over 10^5^ s. These superconducting switches open new possibilities for increasing the size and complexity of modern cryogenic technologies.

Superconducting circuits, thanks to their fast response and low dissipation, have become pivotal to a wide range of computing and sensing applications such as superconducting qubits and quantum processors,^1,2^ infrared single-photon detectors,^3^ and superconducting neurons.^4,5^ However, scaling up the current technologies into practical superconducting systems is hampered by the heat load of processing and readout electronics and the limited available cooling budget of the cryogenic coolers.^6^ Thus, developing low-power-consumption electronics has become increasingly attractive as it can enhance the information processing capacity of superconducting quantum computers and sensors.

A great research effort has been invested in developing compatible cryogenic logic gates and memory cells, which are the building blocks of computing circuits, such as single-flux-quantum (SFQ) technology^7−11^ or nanocryotrons (nTrons).^12,13^ Various technologies that realize these building blocks all have their combinations of scaling challenges such as external magnetic field sensitivity,^14,15^ need for additional timing circuits,^16,17^ large cell dimensions,^18,19^ destructive readout schemes,^13^ insufficient operation speeds,^13,20^ etc. As a result, there remains a demand for new technological concepts to realize both logic gates and memory cells for large-scale, complex cryogenic technologies. For instance, electric-field-controlled cryotrons (EF-Trons)^21−24^ have attracted significant scientific interests due to potential low energy dissipation and high switching frequency, but further studies are required in the dynamic performances and the reduction of the gate voltage.^25,26^ Another promising candidate is the superconducting thermal switch where the superconductivity is perturbed by the Joule heating effect from a heater, such as superconducting nanotransistors (SNTs),^27^ planar heater-nanocryotrons (P-hTrons),^20^ and multilayer heater-cryotrons (M-hTrons),^28^ due to the absence of the leakage between the heater and the superconducting channel. Previous works have shown the potentials of P-hTrons and M-hTrons with DC measurements^27,28^ and electro-thermal simulations.^28^ This work addresses the gap in dynamic characterization and the experimental realization of basic logic and memory cells based on superconducting thermal switches.

In this article, we characterize the static and transient responses of a novel superconducting thermal switch actuated by a metal heater on top of a superconducting nanochannel, as shown in Figure 1. We show that under proper operation conditions, the required switching energy of our devices is no more than a few hundred attojoules. Using these compact nanostructures, we successfully implement fundamental logic gates (NOT, NOR, NAND, AND, and OR). Implementation of these gates are significant, as by combining one (e.g., NOR or NAND) or more (e.g., AND/OR and NOT) types of logic gates, one can realize a complete set of logic operations. We also construct a volatile memory cell with a single superconducting thermal switch based on the persistence of the resistive state in the superconducting channel due to the self-heating effect.^29^ While superconducting nanowire memory cells based on flux trapping have been demonstrated using hTrons,^28,30^ the technology presented here is ultracompact (∼10^–1^ μm^2^) and fabrication-friendly. Both the logic and the memory devices achieve fast operation (up to 100 MHz), outstanding energy efficiency (a few femtojoule per operation), and high reliability (10^–8^∼10^–6^ bit error rate). Therefore, these compatible cells offer a pragmatic approach to developing scalable and energy-efficient cryogenic electrical systems.

**Figure 1:**
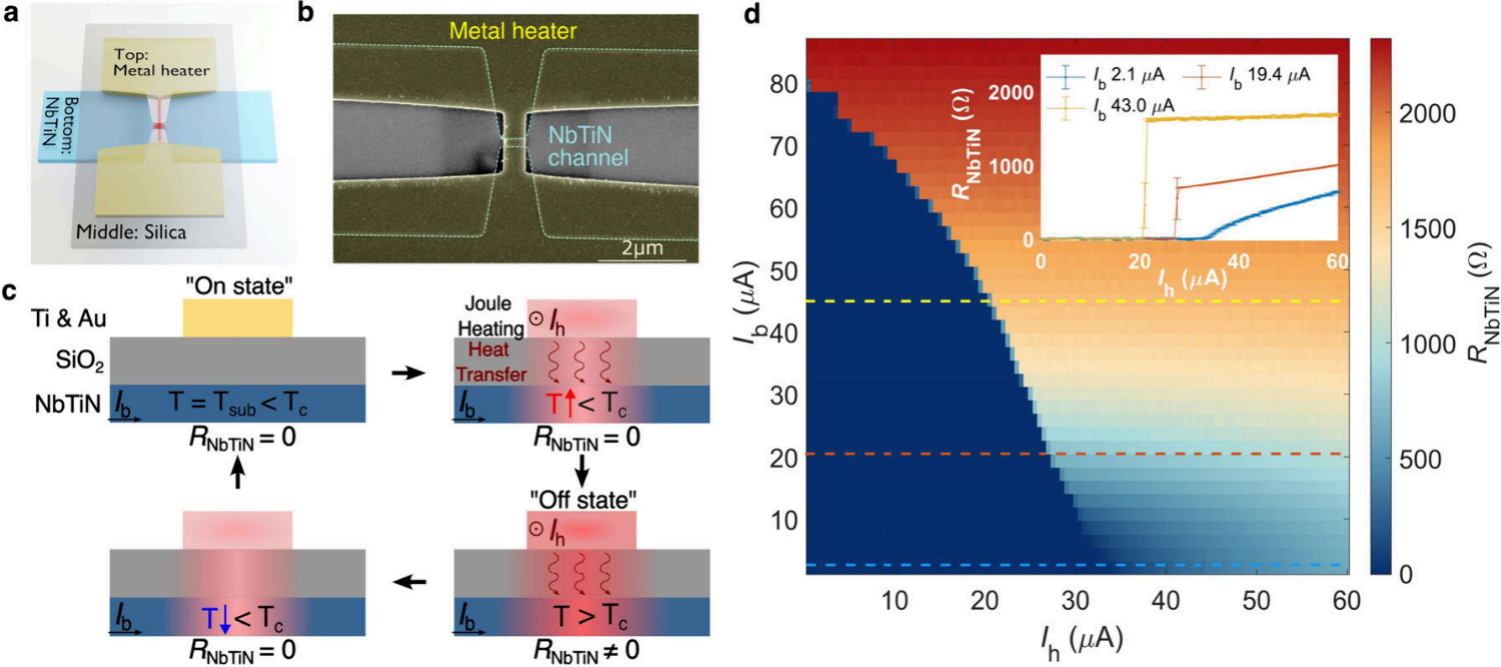
Characterization of the superconducting thermal switch. **a,** Schematic picture and **b,** scanning electron microscopy image of the superconducting thermal switch consisting of a NbTiN channel covered by a layer of silica and a metal heater. **c,** The device switches between the superconducting (“On”) state and the resistive (“Off”) state due to heat transfer from the metal layer via the silica to the NbTiN channel. **d,** Resistance of the NbTiN channel (*R*_NbTiN_) as a function of the bias current (*I*_b_) and the current through the metal heater (*I*_h_).

The structure of the superconducting thermal switch is depicted in Figure 1a. A metal (Ti and Au) heater is placed over a superconducting (NbTiN of 8–10 nm thickness) nanochannel with a thin insulating (SiO_2_) layer in between. As illustrated in Figure 1c, the local heat profiles are determined by Joule heat generation in the metal heater and phonon transport through the SiO_2_ layer. As the local temperature exceeds the critical temperature *T*_c_, the superconducting state of the NbTiN channel is disrupted, resulting in a resistive section (“Off state” in Figure 1c). The resistance in the NbTiN channel is determined by the area of the resistive region, which is related to both the current through the heater, *I*_h_, and the bias current of the NbTiN channel, *I*_b_.

Devices were fabricated as detailed in Supporting Information Section 1. The sample was mounted at a base temperature of ∼3 K. We first measured the response of the superconducting thermal switch in the steady state. Direct currents *I*_h_ and *I*_b_ were sent through the metal heater and the NbTiN channel, respectively, and the resistance of the NbTiN channel *R*_NbTiN_ was measured (see Supporting Information Section 2). Figure 1d displays the results for a device with a NbTiN constriction of 0.5 μm (width) × 0.1 μm (length) and a metal heater of 0.1 μm (width) × 0.5 μm (length). The switching current decreases as the bias current increases due to a lower local critical temperature. As observed from the response curves at various bias currents in the inset, an abrupt change occurs at the switching point when the bias current *I*_b_ is high. This indicates that the resistive hotspot rapidly expands over the entire constriction due to the self Joule heat generation in the nanochannel. As reported in superconducting nanostrips,^31^ we also observed hysteretic characteristics in the *I*_h_-*R*_NbTiN_ measurements and the related discussion can be found in Supporting Information Section 4.2.

In order to evaluate the switching behavior of this device, we measured the transient response. A schematic of the measurement circuit is displayed in Figure 2a (a detailed electrical circuit diagram is illustrated in Supporting Information Section 2). When the input pulse amplitude on the heater triggers a local hotspot, i.e., the superconducting thermal switch is in the off state, the resistance of the NbTiN channel *R*_NbTiN_ becomes large enough to redirect the current to the output amplifier, producing a high voltage level at the output. The dynamic switching energy of the device is calculated as *E*_h_ = τ_h_*V*_h_^2^/*R*_h_, where τ_h_ and *V*_h_ are the duration and amplitude of a single input pulse on the heater, respectively.

**Figure 2:**
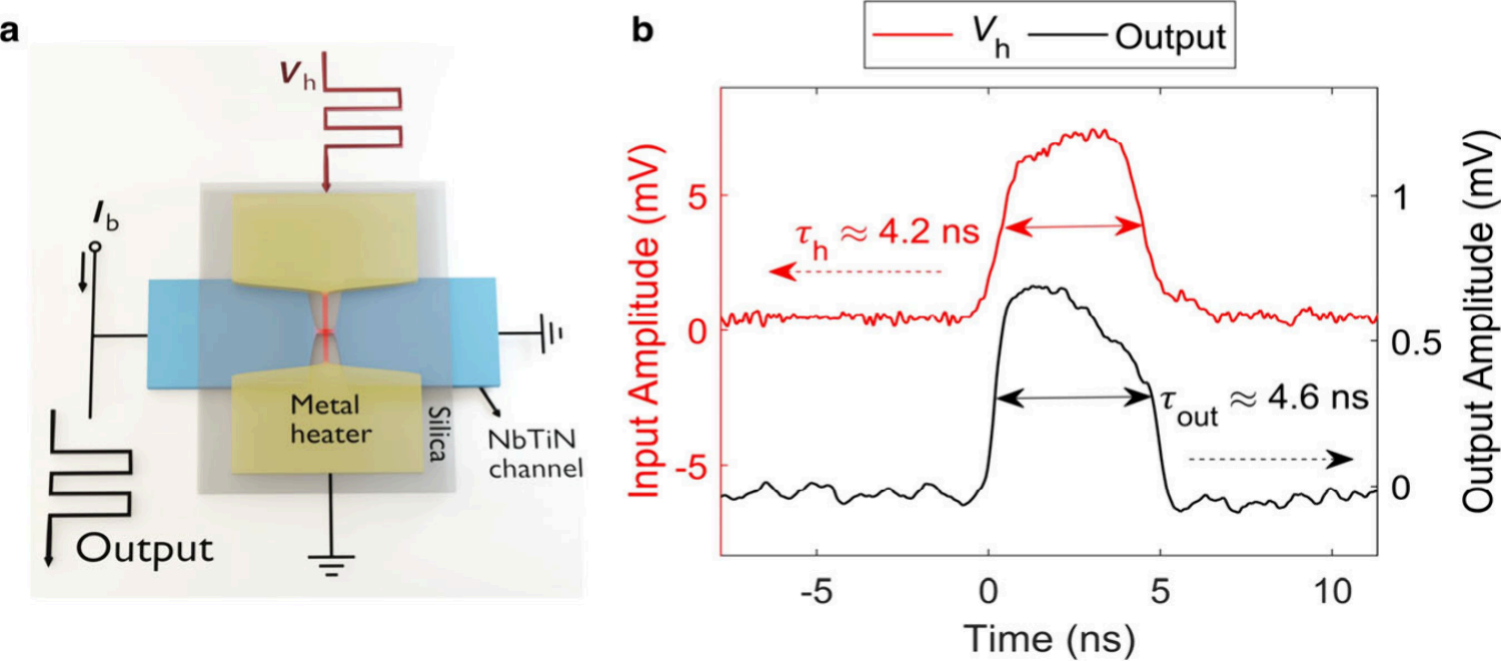
**a,** Schematic of the experimental circuit to test the transient response of the superconducting thermal switch. **b,** Voltage traces of a superconducting thermal switch with a clock input signal. Note: signal attenuations and amplifications have been accounted for, and the corrected curves are plotted (see Supporting Information section 2 for more details). The input signal is a 10 MHz square wave with a pulse duration τ_h_ ≈ 4.2 ns and an amplitude *V*_h_ ≈ 7.2 mV. The device is biased with *I*_b_ = 28.5 μA, and the metal heater has a resistance *R*_h_ = 291.5 Ω.

Figure 2b shows a typical example of the input and output waveforms. It should be noted that the time delay between the input and output signals depends on the length of the associated cables and the differences between electronic components used in the experiment. Here, for easier comparison, the pulse traces are shifted to overlap. The superconducting thermal switch was biased at *I*_b_ = 28.5 μA and could be switched by an input pulse trace with a duration of τ_h_ ≈ 4.2 ns and a frequency of 10 MHz, corresponding to a dynamic switching energy of *E*_h_ = 746.9 aJ. The lowest dynamic switching energy measured was *E*_h_ = 273.8 aJ when the NbTiN channel was biased close to the critical current (see Supporting Information Figure S6a). Such dynamic energy consumption is comparable with that of the modern CMOS switches (100 aJ ∼ 3 fJ).^32,33^ Considering the source-switch impedance mismatches and the connecting transmission lines, the estimated energy consumption presents a higher bound and may be much better (see details in Supporting Information Section 2). Taking into account the energy dissipation due to the current flowing through the channel, the entire switch was estimated to consume 1.658 fJ every switching operation. Further improvement of the energy efficiency can be achieved by scaling down the device dimension to reduce the amount of energy required to break the superconductivity.

The rise and the fall times of the transient response in Figure 2b are 271.8 and 339.1 ps, respectively. The inductor time constant *L*_k_/*R* is not the main limiting factor due to the small NbTiN channel dimensions (*L*_k_ is estimated to be a few nH, see details in Supporting Information Section 1). Constrained by the temporal characteristics of the instrumentation (details in Supporting Information Section 2), the highest switching frequency we experimentally applied was 200 MHz (see Supporting Information Figure S6b). It can be observed that the output pulse duration in Figure 2b does not fully match the input pulse duration. This could be due to the switch-on delay time (see more measurements in Supporting Information Section 6) and the thermal recovery time after the heater switches off. As suggested in previous studies of similar multilayer structures, the switch-on delay could be decreased with a higher input power density or a higher bias current, which influences how fast the channel can be thermalized to the critical temperature.^20,34^ The thermal recovery time is affected by the self-heating effect in the superconducting channel after deactivation of the heater. Apart from engineering the material properties to obtain better thermal coupling between the channel and the substrate, a feasible way to alleviate it is to select a proper constriction geometry as well as an appropriate electrical bias level to reduce the self-heating power density.^35^

By controlling the resistance of the NbTiN channel, we designed a set of fundamental logic gates, e.g., NOT, NOR, NAND, AND, and OR gates. Figure 3 shows the electrical circuits and normalized experimental results for NOT, NOR, and NAND gates. More examples can be found in Supporting Information, Figure S9.

**Figure 3:**
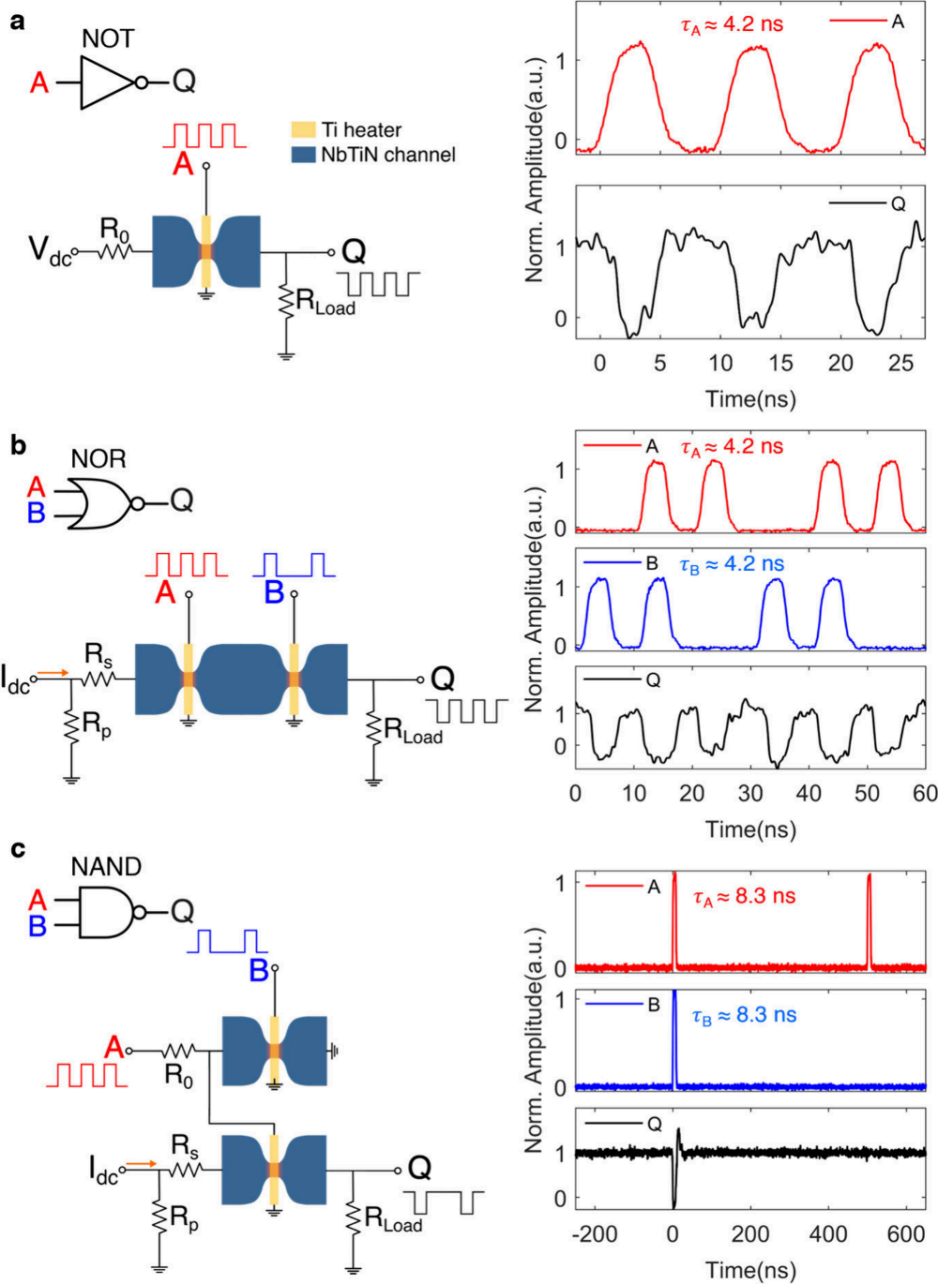
Construction of fundamental logic gates. **a-c,** Implementation of fundamental logic gates NOT (a), NOR (b), and NAND (c) using superconducting thermal switches. Figures on the left are the schematic diagrams. Figures on the right are the corresponding experimental waveforms of the inputs (A, B) and output (Q), rescaled according to the logic HIGH and LOW levels. The resistive elements shown in the figures are *R*_0_ = 22 Ω, *R*_Load_ = 50 Ω, *R*_p_ = 100 Ω, and *R*_s_ = 50 Ω.

In the NOT gate, a voltage source *V*_dc_ is biased through the NbTiN channel of the superconducting thermal switch, in series with a 50 Ω load (*R*_Load_ in Figure 3a). When the voltage across the metal heater is in the logic HIGH level, the NbTiN channel is switched to the nonsuperconducting state, introducing a nonzero resistance (usually ∼ kΩ) in the circuit. Therefore, the current through the load resistor is reduced when the superconducting thermal switch is off, leading to the logic LOW level at the output, as shown in Figure 3a. The bias voltage results in a power dissipation of *V*_dc_^2^/(*R*_0_+*R*_Load_) ≈ 13.9 nW in the on state. Since the normal resistance generated in the NbTiN channel is on the order of a few hundred Ohm according to the static measurement, the energy dissipated in the channel and the bias circuit in the off state is negligible in comparison with the heater input energy. Therefore, the total off-state energy dissipation is around 1.43 fJ.

To implement an NOR gate, two superconducting thermal switches were connected in series (Figure 3b). Once either of the NbTiN channels switches to the resistive state, more current is redistributed to the parallel resistor *R*_p_, leading to a logic LOW level across the output resistor *R*_Load_. Since both switches have significantly larger normal-state resistances than the parallel resistor, there is only a minor difference between the low voltage levels with only one switch or both switches in resistive state. Compared with the NOT gate, a higher static power (∼54.5 nW) was dissipated due to the resistor bridge in the on state. The energy dissipation in the biasing and observation circuit is approximately 0.457 fJ, and the input heating pulse energies on the two heaters are 1.54 fJ and 1.53 fJ, respectively. Thus, the resulting energy dissipation of the whole NOR gate in the off state is around 3.53 fJ.

Analogously, one can assemble two superconducting thermal switches in parallel to build a NAND gate. This implementation requires an appropriate gate threshold or a delicate circuit design (Supporting Information Figure S10). Figure 3c represents another more straightforward way to construct a NAND gate by connecting a NOT gate with an AND gate. Thanks to the insulation between the superconducting channel and the heater, the bias current and the heater current can be modulated simultaneously without considerable crosstalk. Therefore, the heater of a superconducting switch can be driven by the input signal B, while the bias current through the superconducting channel can be controlled by the other input signal A, forming an AND gate. The overshoot at the output, which can be observed in Figure 3c (as well as for the two-stage logic circuit in Figure 4b), can be attributed to the off-chip connection using coaxial cables between the two stages in the initial experiments. During the switching-off time of 8.3 ns, the energy dissipated in the NAND gate is about 18.83 fJ. It can be further decreased by using a voltage source for the NOT gate to avoid the static power consumption in the resistor bridge, integrating elements completely on-chip to increase the signal transfer efficiency, or downscaling the superconducting thermal switch to achieve less energy dissipation.

**Figure 4:**
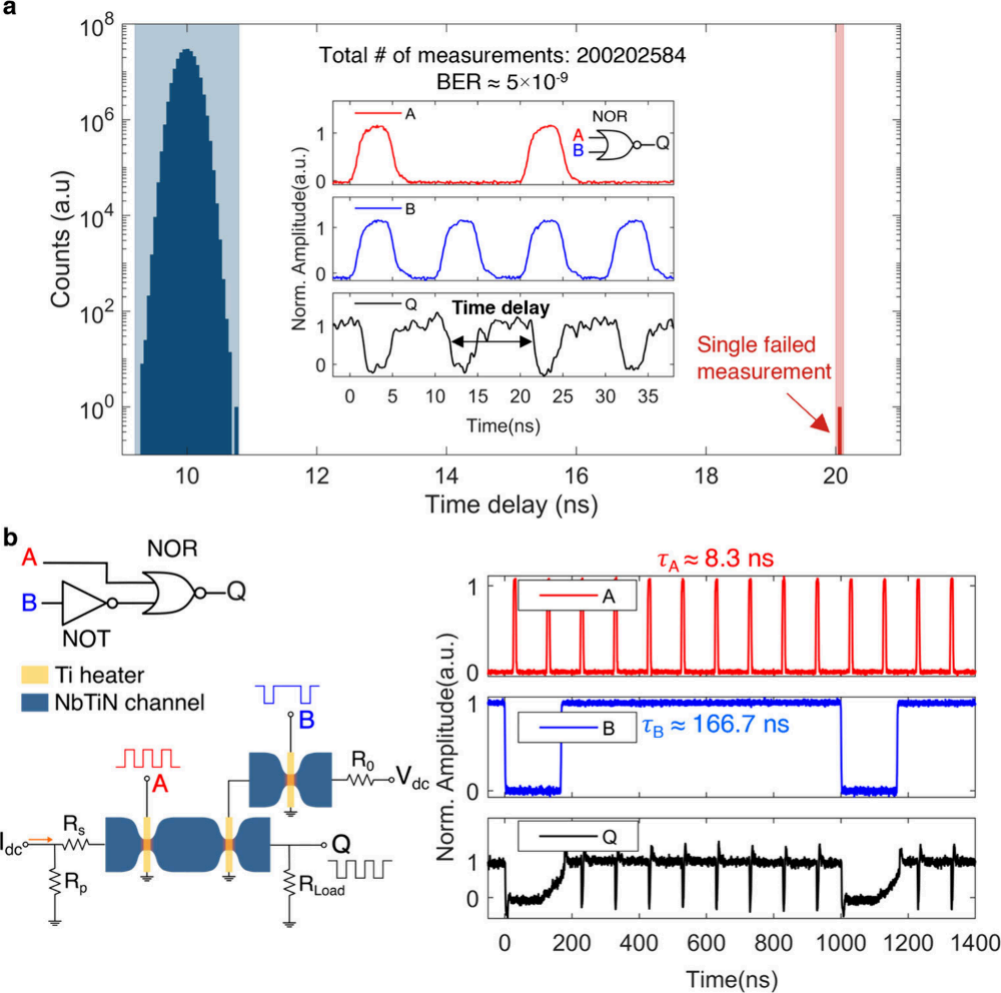
Bit error rate (BER) and driving ability of the logic gates. **a,** Correct (blue) and incorrect (red) operations of a NOR gate over 2 × 10^8^ measurements as used to calculate the bit error rate (BER). The inset displays typical input (A: 50 MHz with a pulse duration of 4.2 ns, B: 100 MHz with a pulse duration of 4.2 ns) and output signals (Q) of the NOR gate. The horizontal coordinate represents the time delay between the falling edges of two consecutive pulses at the output, which was triggered at 50% of the output pulse amplitude. **b,** Electrical diagram (left) and experimental results (right) of a simple logic circuit composed of a NOT gate and a NOR gate.

One crucial merit of any logic gate is the bit error rate (BER), which is defined as the number of incorrect responses at the output divided by the total number of input events. We achieved a BER of 5 × 10^–9^ for the NOR gate operation, as shown in Figure 4a. The two superconducting thermal switches were operated with input pulse energies of 1.40 (input A) and 1.18 fJ (input B). The single fail event among over 200 million measurements was detected by comparing the time delay between the two consecutive output pulses with the supposed period of the output signal, which was 10 ns for Figure 4a.

The construction of NAND gates by combining NOT and AND gates suggests that the superconducting thermal switches can drive consecutive logic gates, which is important in building more complex superconducting logic circuits. To further explore this, we constructed the circuit shown in Figure 4b, where a NOT gate and an NOR gate are connected to form a simple logic circuit in such a way that one gate drives the input (heater) of the next stage. The determining factor to trigger the heater in the next stage is to generate a HIGH voltage level exceeding its switching threshold. Since the switch in the NOT gate is supposed to stay in the superconducting state at logic HIGH output, the HIGH voltage level is mainly constrained by the critical current of the NOT gate. Thus, the superconducting thermal switch used in the NOT gate was designed to have a wider NbTiN channel in order to provide enough switching energy to the next stage.

Recently superconducting memory cells driven by both optical and electrical signals have been demonstrated using the hysteretic transition between the superconducting and the resistive state.^29^ These devices consume ∼600 μW power in the resistive state and operate at speeds <50 kHz. In Figure 5, we present a novel approach to construct a volatile memory cell with a superconducting thermal switch, which allows a higher speed and lower energy consumption (see Supporting Information Figure S11 for alternative implementations).

**Figure 5:**
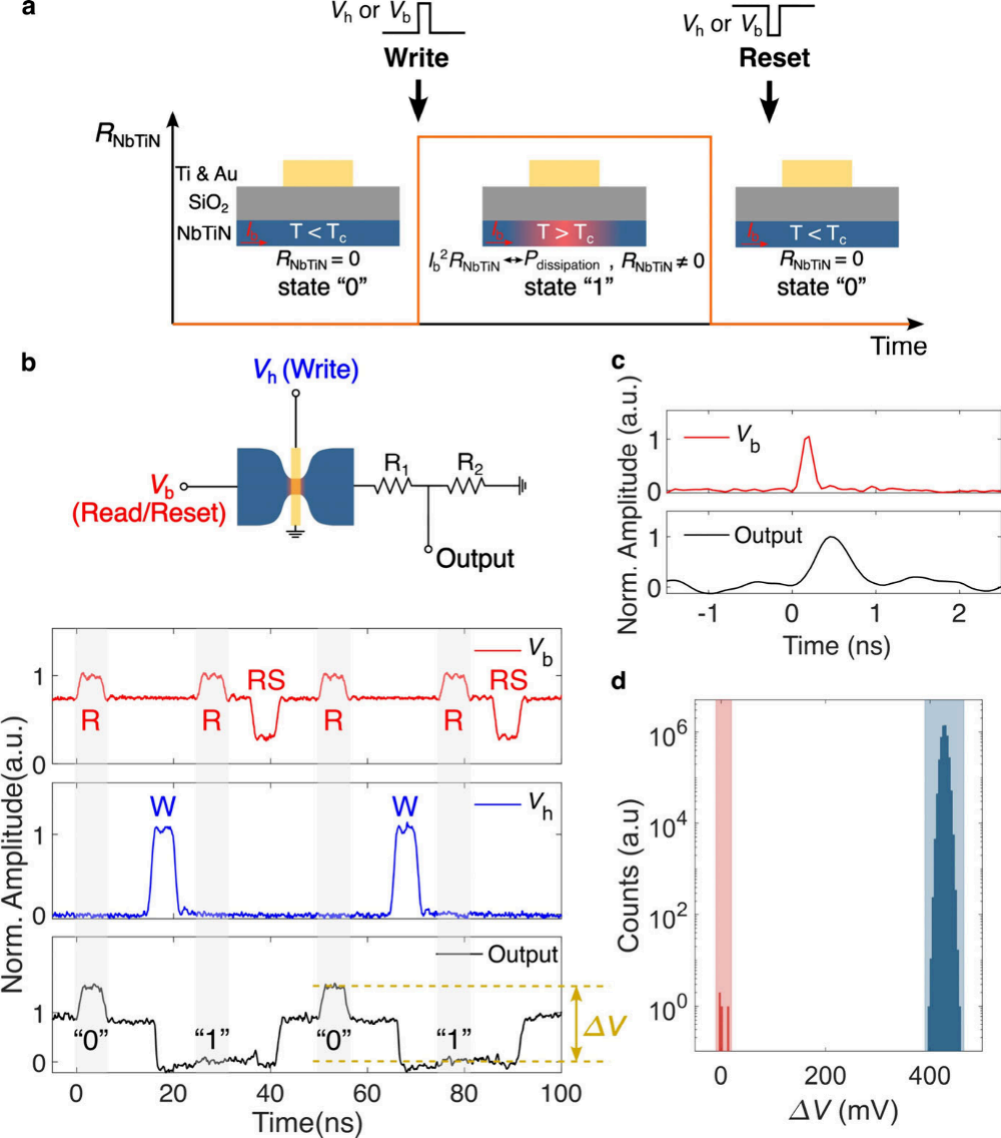
Memory operations of a superconducting thermal switch. **a,** Use of the superconducting thermal switch as a memory cell. **b,** Circuit diagram (top) and experimental characteristics (bottom) of the memory. The output Q is amplified by an AC-coupled amplifier (50 Ω input impedance). *R*_1_ = 22 Ω and *R*_2_ = 50 Ω are chosen for better impedance matching. Basic memory operations (bottom) are denoted as W for Write, RS for Reset, and R for Read (“0” for reading a superconducting state, where an output pulse is captured, and “1” for reading a resistive state, where no output signal is observed). **c,** Read operation of the state “0” with a subnanosecond input pulse. The output pulse has a fwhm of around 550 ps. **d,** Measurement of the BER based on the output voltage difference Δ*V* between “0” and “1” state. The 4 incorrect measurements are marked in red, and correct measurements in blue. The total number of measurements is five million, leading to a BER of 8 × 10^–7^.

As depicted in Figure 5, the channel in a superconducting thermal switch can be intentionally latched into the nonsuperconducting state (state “1” in Figure 5a), if the electro-thermal effect in the superconducting channel and the heat dissipation into the substrate reach an equilibrium. By reducing the power dissipated from the channel or the heater to alleviate the electro-thermal effect, the device can be reset back to the superconducting state (state “0” in Figure 5a).

In the example shown in Figure 5b, the Write or Reset operations are realized by increasing the heater voltage *V*_h_ or decreasing the bias voltage *V*_b_. The “0” and “1” states of the device are distinguished based on the response at the output by sending a small pulse through the channel. When reading the resistive (“0”) state, the output voltage level remains stable since *R*_NbTiN_ ≠ 0 is dominant compared to the associated resistance, resulting in a negligible variation in the current flowing through the circuit; when reading the superconducting (“1”) state, a pulse can be observed at the output due to *R*_NbTiN_ = 0.

The memory could retain the states for over 10^5^ s (see Supporting Information Figure S12). Speeds of up to 80 MHz were reached, which are a limit set by our instruments and do not represent the upper limit of our device. The Read operation reached subnanosecond speeds (Figure 5c), and the Write operation could be completed within ∼5 ns (Figure 5b). A BER of 8 × 10^–7^ was measured with more than 5 million consecutive Read-Write-Read-Reset operations. The memory consumes 56.2 nW in the superconducting state (state “0”), and ∼20 nW in the resistive state (state “1”). The energy dissipation was around 1 fJ on the heater to write “1” and ∼625 aJ in the channel to read “0”. While the mentioned values are comparable with the state-of-the-art nTron memory and logic devices,^13^ our technology additionally offers nondestructive readout and a small area (∼10^–1^ μm^2^), allowing for a high integration density (see Supporting Information Table S1 for an overview).

In conclusion, we demonstrated a superconducting thermal switch that takes only attojoules to trigger and has a total energy consumption on the order of femtojoules. The multilayered structure is fabrication-friendly and can be scaled down further. Utilizing these superconducting thermal switches, we successfully constructed high-fidelity fundamental logic gates. Compared with standard CMOS logic gates,^36^ this superconducting logic family requires fewer switching elements and consumes only femtojoules of energy per operation. Furthermore, we realized Set-Reset memory operations using a single superconducting thermal switch with a low error rate on the order of 1 × 10^–7^ and a nanosecond operation speed.

The compactness and reliability of these logic gates is attractive for cryogenic applications, such as superconducting nanowire single-photon detectors (SNSPDs). SNSPDs are the main choice for optical signal detection in applications such as quantum communication,^37^ due to their exceptional detection efficiency^38,39^ and low time jitter,^40^ but are difficult to combine into large arrays due to the complexity of their readout circuitry.^41−43^ As the cryostat provides ∼100 mW cooling budget at 3 K and a single superconducting thermal switch can reach a power consumption <1 μW, around a hundred thousand of current elements are afforded in the cryogenic system to construct digital circuits. Thus, it is a promising route to develop novel readout techniques by integrating SNSPDs with the compact, energy-efficient, and fabrication-friendly superconducting electronic components proposed here. Adjusting the device geometries and superconducting parameters can further improve the power efficiency and hence the integration capacity. Furthermore, the superconducting logic gates and memory cells have the potential to be utilized in high-performance cryogenic digital processors^44−47^ as well as hybrid circuits that combine semiconducting and superconducting components.^34,48^

## Supporting Information Available

Fabrication process, electrical setup, additional measurements about switching behaviors, thermal crosstalk, switch-on latency, alternative approaches to realize logic or memory operations, and comparison with other superconducting devices (PDF)
