# Supplementary material for: Attojoule Superconducting Thermal Logic and Memories
Source: Nano Lett. 2025 Mar 10;25(11):4401–7. doi: 10.1021/acs.nanolett.4c06545 (PMC11926957; doi:10.1021/acs.nanolett.4c06545)
Supplement: Supplementary file 1 — nl4c06545_si_001.pdf [file nl4c06545_si_001.pdf]

Attojoule superconducting thermal logic and memories: Supporting Information

Hui Wang[1], Niels Noordzij[2], Mischa Mikhailov[1], Stephan Steinhauer[3], Thomas Descamps[3], Eitan Oksenberg[2], Val Zwiller[3] & Iman Esmaeil Zadeh[1,2]

[1] *Department of Imaging Physics, Delft University of Technology, 2628CN Delft, The Netherlands.*
[2] Single Quantum B.V., 2628 CH Delft, The Netherlands.

[3] Department of Applied Physics, Royal Institute of Technology (KTH), SE-106 01 Stockholm, Sweden

Contents

1. Sample Fabrication 2
2. Electrical set-up 2
3. Switching power density 4
4. The static and dynamic performances of superconducting thermal switches 5
   4.1 Comparison of the static responses of two superconducting thermal switches 5
   4.2 Hysteresis measurement 6
   4.3 Supplements of AC measurements 6
5. Thermal crosstalk of the device 7
6. Latency of the superconducting thermal switch 8
7. OR and AND gates 9
8. NAND gate implementation 10
9. Memory cell implementations 11
10. Retention time measurement of the memory cell 12
11. Comparison with literature values 12
12. Supplementary figures without amplitude normalization 14

1. Sample Fabrication

NbTiN films are deposited by magnetron co-sputtering in an Ar and N_2 atmosphere on thermally oxidized silicon wafers. The thickness of the NbTiN films were in the range of 8-10 nm, leading to a film critical temperature of 9-11 K (more information about the film deposition and the critical temperature measurement can be found in the previous study[1,2]). The sheet inductance of the film is estimated to be L_{sheet} =100 pH/□. Taking into account the geometry of both the bonding pads and the superconducting channel, the kinetic inductance of our device is approximately $10\sim20L_{\text{sheet}}$, which is in the order of a few nH.

We first create a lift-off pattern with electron-beam lithography (EBL, acceleration voltage of 100 kV) using a double-layer PMMA resist for the gold contact pads. After development in a solution of MIBK:IPA=1:3, a 5 nm-thick Cr layer (as the stitching layer) and a 55 nm-thick Au layer are deposited with E-beam metal evaporation (Temescal FC2000) and then the sample is lifted off in Acetone at 45 °C. Secondly, the NbTiN structures are patterned with EBL using a positive e-beam resist ARP6200.4. Afterwards the resist is developed using pentyl acetate and a solution of MIBK:IPA=1:1. Then the NbTiN layer is etched by Reactive Ion Etching with SF_6 and O_2. Third, the whole sample is covered with a silicon dioxide layer of 40 nm thickness deposited with ICPCVD (Inductively Coupled Plasma Chemical Vapor Deposition) or PECVD (Plasma Enhanced Chemical Vapor Deposition) at 300 °C. This layer primarily serves as an insulating layer but also protects the NbTiN structures from oxidization. Lastly, the metal heater and its contact pads are patterned in another lift-off process with the same double-layer PMMA resist. The metal heater is composed of an 80 nm-thick Ti layer and a 5 nm-thick Au layer to reduce the heater resistance.

2. Electrical set-up

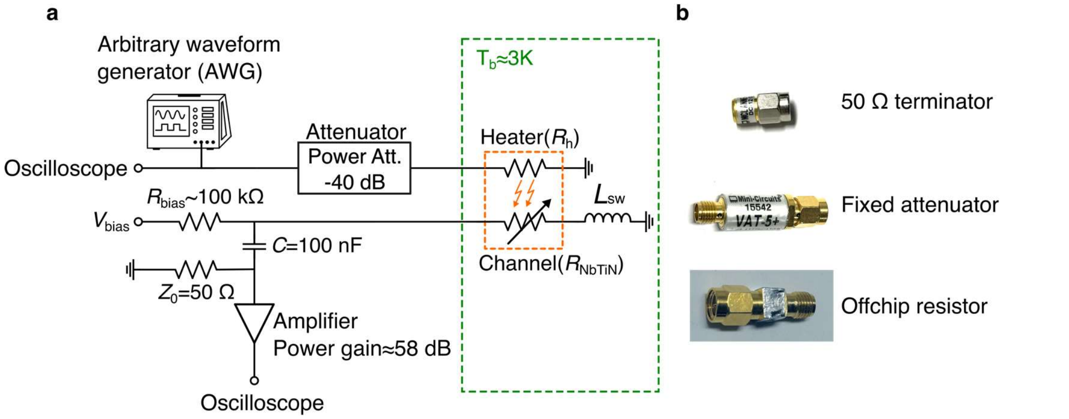

Figure S1. Electrical set-up of the measurement. **a,** Schematic of the electrical circuit for the AC measurement of a single superconducting thermal switch, which was used for Figure 2b. **b,** Images of the off-chip components, including 50 Ω terminators, fixed attenuators, and manually soldered resistors with SMA connectors.

The sample was fixed within a commercial Gifford-McMahon cryocooler to achieve a base temperature of around 3 K. The electrical sources and measuring units were connected to the sample externally via coaxial cables, whose characteristic impedance is 50 **Ω**. It should be noted that the impedance mismatch between the superconducting thermal switches and the coaxial cables can influence the signal transfer efficiency in AC measurements.

The direct current source used in all the electrical measurements consisted of a voltage source and a large bias resistor (usually ~100 kΩ, as shown in Figure S1a), so that the driving current was approximately constant regardless of the load impedance (usually from 0 to a few kΩ). In the static measurement of the superconducting channel resistance R_{NbTiN} in Figure 1d, the bias current I_{b} and the heater current I_{h} were driven with two separate current sources. Then we obtained R_{NbTiN} by measuring the voltage drop on the NbTiN channel V_{NbTiN} and using $R_{\mathrm{NbTiN}} = V_{\mathrm{NbTiN}}/I_{\mathrm{b}}$.

Regarding the AC measurements, the measurement scheme for Figure 2 (main text) is depicted in Figure S1a as an example. The input pulses and the DC voltage signals, in Figures 2-5 of the main text, were generated with an arbitrary waveform generator (AWG: Tektronix AWG5014C, sampling rate 1.2 G samples/s, and Siglent SDG6032X, sampling rate 1.2 G samples/s) or a short pulse generator (Figure 5c: Highland Technology Model T240). They were attenuated at room temperature with attenuators from Mini Circuits (typically 40 dB power attenuation (100× voltage attenuation), by combining several fixed attenuators shown in Figure S1b) and subsequently sent to the integrated heater via coaxial cables, a PCB, and bonding wires. The sampling rate of the arbitrary waveform generator limits the rising and the falling time of the input signals to ~800ps. Here the input impedance of the amplifier Z_0 = 50 Ω was considered as the load impedance of the switch. The amplifier was AC-coupled via a capacitor of 100nF. It has a power gain of ~58 dB and a bandwidth of ~2 GHz. All waveforms were captured with a Teledyne LeCroy WaveRunner 640Zi oscilloscope, with sampling rates of 20 or 40 G samples/s (4 GHz analog bandwidth). To build electrical circuits described in Figure 3, 4 and 5 in the main text, 50 Ω terminators or manually soldered resistors with SMA connectors (see Figure S1b) were connected outside of the cryostat via coaxial cables. Further integration of the superconducting switches and the resistors on chip will be one of our focuses in future experiments.

The bit error rate (BER), which is defined as the ratio between the number of false events and the total number of events, was measured by creating histograms of the output signals in response to periodic features in the inputs. The output histogram was then compared with the expected results. In Figure 4a, for example, the output of the NOR gate was a periodic signal with a frequency of 100MHz. Using the internal function of the oscilloscope to measure the time delay between two adjacent falling edges (trigger level is 50% of the amplitude), the correct events resulted in a time delay of around 10 ns while a false event lead to a time delay of ~20 ns. The histogram function which is built in the oscilloscope allows us to record the results of the time delay measurement, as Figure 4a displays. Thus, the BER in Figure 4a was determined as 1/ 200202584 ≈ 4.99×10^{-9}. In the other BER measurement in Figure 5d, the status of the memory cell was read after every Write or Reset operation many times and histograms were created and compared with expected values. We utilized the measurement functions in the oscilloscope to obtain the voltage levels when reading “0” state and reading “1” state, respectively. The voltage difference ΔV was then calculated. If ΔV was close to zero, the device was not properly written/reset in the cycle, which corresponded to the four false events marked in red in Figure 5d. Since we collected the data for 5×10^{8} cycles of Write-Reset operations, the resulted BER is 4/ 5×10^{8} ≈ 8×10^{-7}.

In order to estimate the energy consumption of the device, the resistance of the heater was measured with a handheld multimeter outside the cryostat, which leads to an overestimation due to the resistance of the bonding pads and the connections between heaters and multimeter probes. To analyze the influence of the resistance of bonding pads, we implemented a 3-wire measurement of a metal heater with a length of 1 μm and a width of 100 nm (Figure S2a). Two

bonding pads on the left are connected to the ground while the other two on the right are connected to two voltage monitors, V_1 and V_2, respectively. The resistances are determined by the linear fitting of the IV curves. The result in Figure S2b indicates that around 40 % of the total resistance is attributed to one bonding pad. Therefore, the power/energy consumption of the device can be optimized in further experiments by reducing the bonding pad resistance such as fabricating the bonding pads with a material with a lower resistivity or increase the layer thickness on the bonding pads.

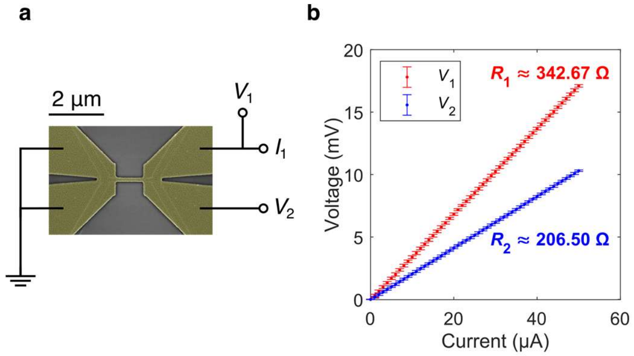

Figure S2. 3-wire measurement of the metal heater. **a,** Schematic of the measurement setup. **b,** Results of the IV measurement with V_1 and V_2.

3. Switching power density

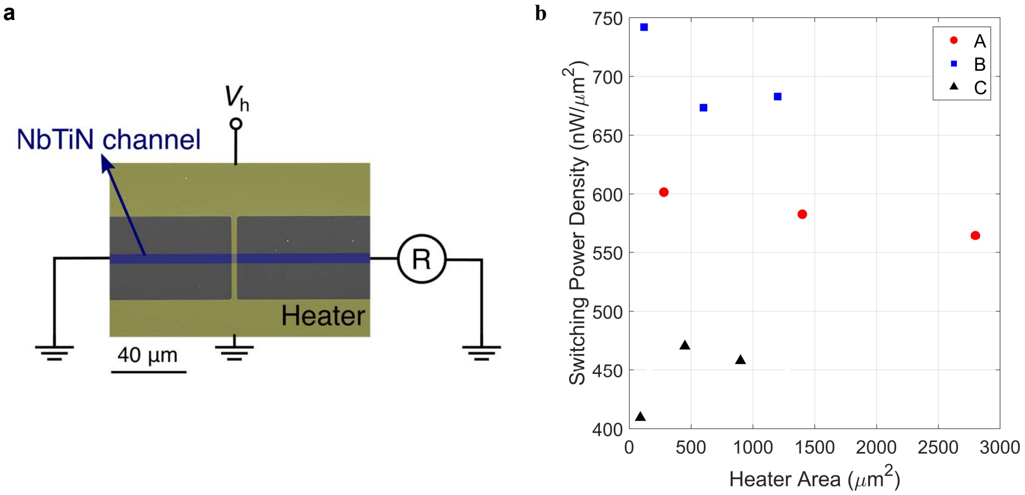

Figure S3. Switching power density measurements. **a,** Measurement set-up to analyze the switching power density of the superconducting thermal switches. **b,** Experimental results for 9 devices on the same sample, categorized into groups A, B, and C according to the superconducting channel width (A: 100 µm, B: 20 µm, and C: 5 µm).

To study the variation in the switching power density of a NbTiN film, several superconducting thermal switches were fabricated on one sample. Each device consisted of a straight superconducting channel with a width w_{NbTiN}, and a metal heater with an area $w_{\mathrm{H}} \times l_{\mathrm{H}}$, as schematically displayed in Figure S3a. In the Figure, they are grouped into three categories

A, B, and C, according to the superconducting channel width w_{NbTiN}, which was 100 μm, 20 μm, and 5 μm, respectively. A Keithley 2000 multimeter was used to monitor the transition of the superconducting channel using the four-terminal resistance-sensing mode. The influence of the bias current provided by the Keithley on the switching behavior could be neglected since it was around 7 μA, which was much smaller than the critical currents of the superconducting channels. As shown in Figure S3b, devices in the same group switch at a similar switching power density. It can be observed that discrepancies exist among the three groups, which may be attributed to fabrication inaccuracies and variations in the local thermal conduction between the substrate and the cooling stage.

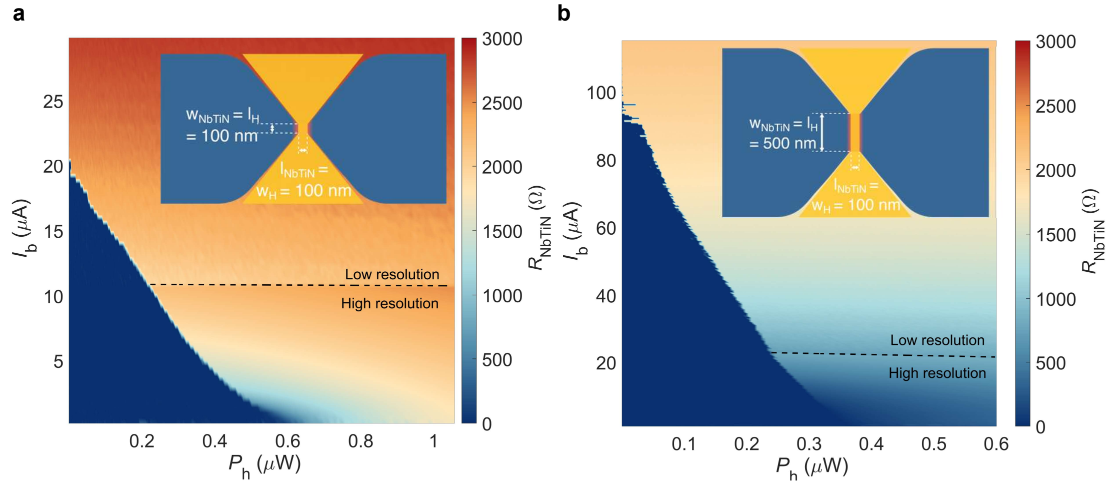

Figure S4. Direct-current characteristics of the superconducting thermal switch. **a** and **b,** Static channel resistance R_{NbTiN} as a function of the power dissipated by the heater $P_{\mathrm{h}} = I_{\mathrm{h}}{}^{2}R_{\mathrm{h}}$ and the bias current through the channel I_{b} for two different superconducting thermal switches. The device dimensions are shown in the insets.

4. The static and dynamic performances of superconducting thermal switches

4.1 Comparison of the static responses of two superconducting thermal switches

Figure S4 shows the static measurements of the channel resistance R_{NbTiN} of two superconducting thermal switches of different sizes (indicated in the insets of the Figure) fabricated on the same chip. As mentioned in the Methods section, the channel resistance is calculated according to $R_{\mathrm{NbTiN}} = V_{\mathrm{NbTiN}}/I_{\mathrm{b}}$. Given the limited measuring range of the voltmeter, the results are a combination of data acquired with a high-resolution voltmeter at low bias currents and a low-resolution voltmeter at high bias currents. In both figures, the switching heater power at the transition from the superconducting to resistive state decreases as the bias current I_{b} increases. The switching heater current becomes unstable as I_{b} approaches the critical current of the NbTiN channel, which is probably due to thermal fluctuations within the set-up. When comparing the switching heater power at a small bias current that is negligible compared to the critical current, it becomes clear that the device in Figure S4a demands a higher switching power than the one in Figure S4b, although the heater size is smaller. However, such a comparison may not be completely meaningful unless the influence of the bonding pads and the connecting wires, which link the critical heater structure to the electrical source, is excluded. To accurately assess the switching power consumption of

the superconducting thermal switch, the bonding pads can be made of NbTiN instead of Ti in future experiments. Besides, the difference in switching power could also be attributed to fabrication non-uniformity.

4.2 Hysteresis measurement

It is essential to evaluate the hysteresis of the superconducting thermal switches, which provides an insight about the working conditions for the logic or the memory operations. The hysteresis measurement was implemented on a superconducting thermal switch which has a NbTiN channel of 500 nm (width) × 100 nm (length) and a metal heater of 100 nm (width) × 500 nm (length). Analyzing the DC results with the heater current I_h sweeping in the increasing and decreasing directions (as shown in Figure S5a and S5b), we obtained the switching heater current (I_h+) and the retrap heater current (I_h-) of the device in Figure S5c.The switching heater current falls to zero at a bias current of I_b =109 μA while the retrap heater current is reduced to zero with I_b >19 μA. This indicates that the device can memorize the resistive state even after removing the heater current when the bias current I_b >19 μA, which is crucial in the memory operation. On the contrary, as indicated by the green area in Figure S5c, the device can recover back to the superconducting state by decreasing the heater current when I_b <19 μA, corresponding to the situation of the logic gates.

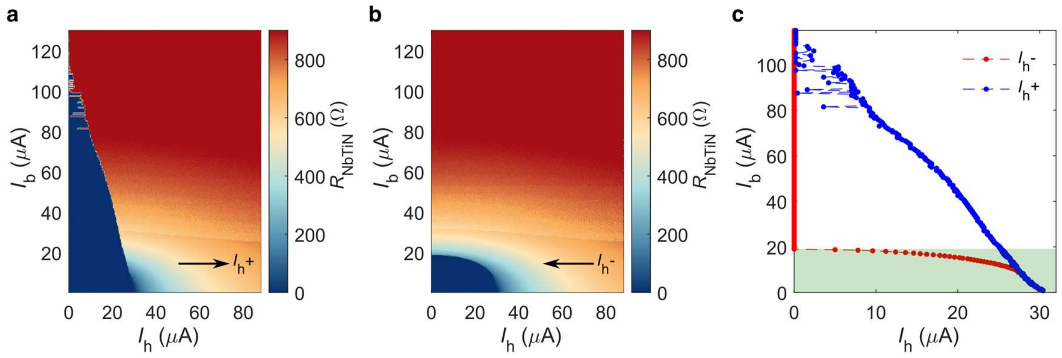

Figure S5. Hysteresis measurement of a superconducting thermal switch with a NbTiN channel of 500 nm (width) × 100 nm (length) and a metal heater of 100 nm (width) × 500 nm (length). **a,** Measurement of the channel resistance R_NbTiN while scanning I_h in the increasing direction. **b,** Measurement of the channel resistance R_NbTiN while scanning I_h in the decreasing direction. **c,** The switching heater current and the retrap heater current of the NbTiN channel obtained from the results in **a** and **b**. The region with non-zero retrap heater currents is shaded in green.

4.3 Supplements of AC measurements

The transient responses of the superconducting thermal switches with various geometries were measured with the same experimental set-up depicted in Figure S1a. Among all the measurements, the lowest dynamic switching energy we achieved was 273.8 aJ (Figure S6a) and the highest switching speed was 200 MHz (Figure S6b).

The device used in Figure S6a has a NbTiN channel of 139 nm (width) × 500 nm (length) and a Ti heater of 100 nm (width) × 3840 nm (length). It was biased with I_b = 31 μA, which was just below its critical current, in order to reach the best dynamic energy consumption of the superconducting switch. According to the input pulse shape (which was attenuated with 20

dB on the heater) and the resistance of the heater $R_{\mathrm{h}} = 594\ \Omega$, the dynamic switching energy measured was $E_{\mathrm{h}} = 273.8$ aJ. The mismatch between the input and the output pulse durations, which might be due to self-heating effects within the NbTiN channel, prevents further investigation into the limit of the switching speed.

Another device tested in Figure S6b has a NbTiN channel of 500 nm (width) × 100 nm (length), and a heater of 100 nm (width) × 500 nm (length). The NbTiN channel has a lower resistance in the normal state, which helps mitigate the self-heating effect and hence increases the switching speed. The experimental results suggest that this device could work at an operation speed of 200 MHz with a pulse duration of 2.5 ns. The Ti heater resistance was $R_{\mathrm{h}} = 282.5\ \Omega$ (100 nm (width) × 500 nm (length)). Thus, the estimated switching energy per operation on the heater was around 580.6 aJ. We believe that the performance of the superconducting thermal switch can be further improved by optimizing the geometries and the electrical circuits.

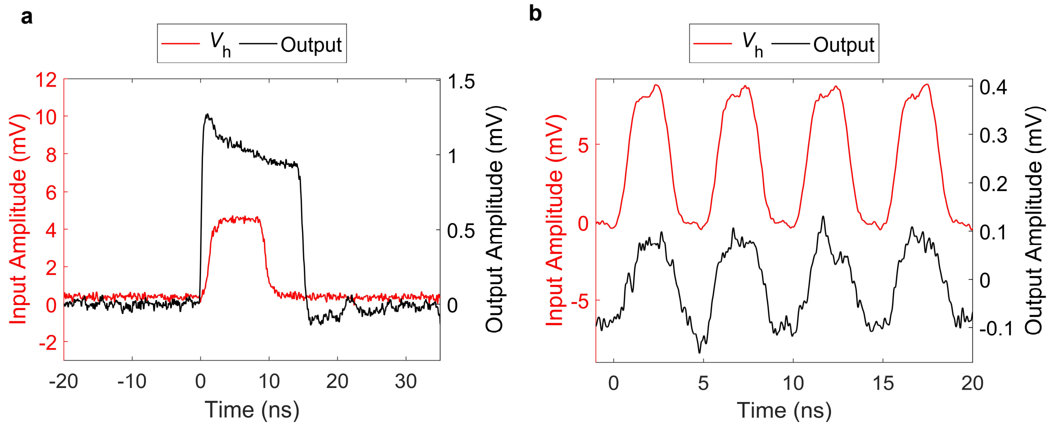

Figure S6. Response of the superconducting switch with the smallest pulse energy and highest switching frequency. Note: signal attenuations or amplifications have been considered and corrected in the figure. **a,** Voltage traces of a superconducting thermal switch with an input pulse energy of 273.8 aJ. The input signal is a 1 MHz square wave with an amplitude of 4.4 mV and a pulse duration $\tau_{\mathrm{h}} \approx 8.33$ ns. **b,** Voltage traces of a superconducting thermal switch operated at 200 MHz. The input signal has an amplitude of 8.1 mV, a pulse duration $\tau_{\mathrm{h}} \approx 2.5$ ns and a duty cycle of 0.5.

5. Thermal crosstalk of the device

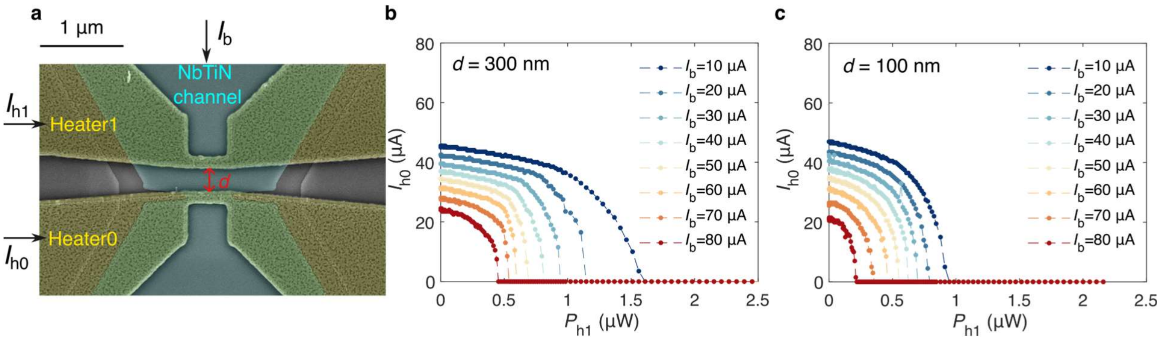

Figure S7. Performance of a superconducting thermal switch with the disturbance of a second heater. **a,** A false-color SEM image of the sample. Heater0 is fabricated on top of the superconducting constriction, and Heater1 is

placed near Heater0 with a distance of d. **b and c,** The switching current through Heater0 (I_{h0}) influenced by the Joule heating power generated by Heater1 (P_{h1}) at a distance of d = 300 nm and d = 100 nm.

Since the thermal effect is not perfectly localised under the heater, there is thermal crosstalk between superconducting thermal switches. To evaluate the crosstalk effect, we designed and fabricated a superconducting switch with two heaters, positioning one heater exactly above the constriction (Heater0 in Figure S7a), and a second heater at a distance d from the first one (Heater1 in Figure S7a). The channel has a width of 500 nm and a length of 100 nm, resulting in a critical current of 110 μA. The two heaters have the same geometry with the size of 100 nm (width) × 500 nm (length) (the resistances of Heater0 and Heater1 are around 350 Ω). The results for heater distances of d = 300 nm and d = 100 nm are shown in Figure S7b, the switching heater current through Heater0 is reduced as the Joule heating power from Heater1 increases. Besides, the higher the bias current, the more sensitive the device is to the thermal crosstalk induced by the second heater. By adjusting the working condition (such as the bias current), the interference caused by the thermal crosstalk can be tolerable for the stability of the device with a distance in the order of 100 nm. Since superconductors are poor heat conductors[3] and the SiO_2 insulating layer mainly supports the lateral heat transfer process, reducing the insulating layer thickness can be helpful to minimize the thermal crosstalk and enhance the integration density in the future.

6. Latency of the superconducting thermal switch

In the previous research of superconducting thermal switches, a dependence between the turn-on delay time of the switching behaviour and the heating power has been reported.[4] We also observed a similar behaviour in our devices. As shown in Figure S8a, an input pulse with a duration of 5 ns at a frequency of 10 MHz was applied on the heater. We measured the latency with the systematic offset, which is defined as the time delay between the rising slopes of the input and the output signal, with the oscilloscope at different input amplitudes V_h for over 450k periods. Due to the lack of measurements on the absolute temporal delay induced by the cables and other electronics, the relative latency compared with the results at V_h=15 mV is shown in Figure S8b. As the input amplitude decreases from 15 mV to 11.8 mV, the relative latency is increased by 1.7 ns. The jitter of the switching behaviour also becomes larger, which can be attributed to the stronger interference of the temperature fluctuation of the cryostat or the electrical noises in the circuit.

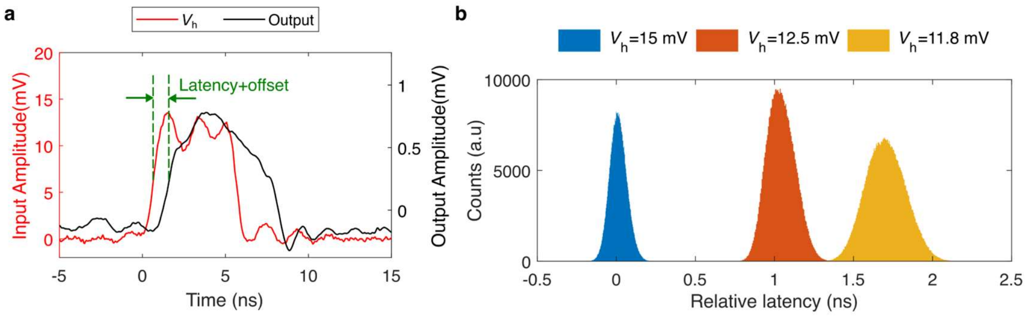

Figure S8. Latency with the systematic offset of the superconducting thermal switch. **a,** An example of the input and the output waveforms in the AC measurement. The input pulse width is 5 ns and the frequency is 10 MHz. The device is biased at 30 μA (the channel with is 500 nm, resulting in a critical current of 109 μA). **b,** Relative latency of the superconducting thermal switch with different input amplitude V_h on the heater.

7. OR and AND gates

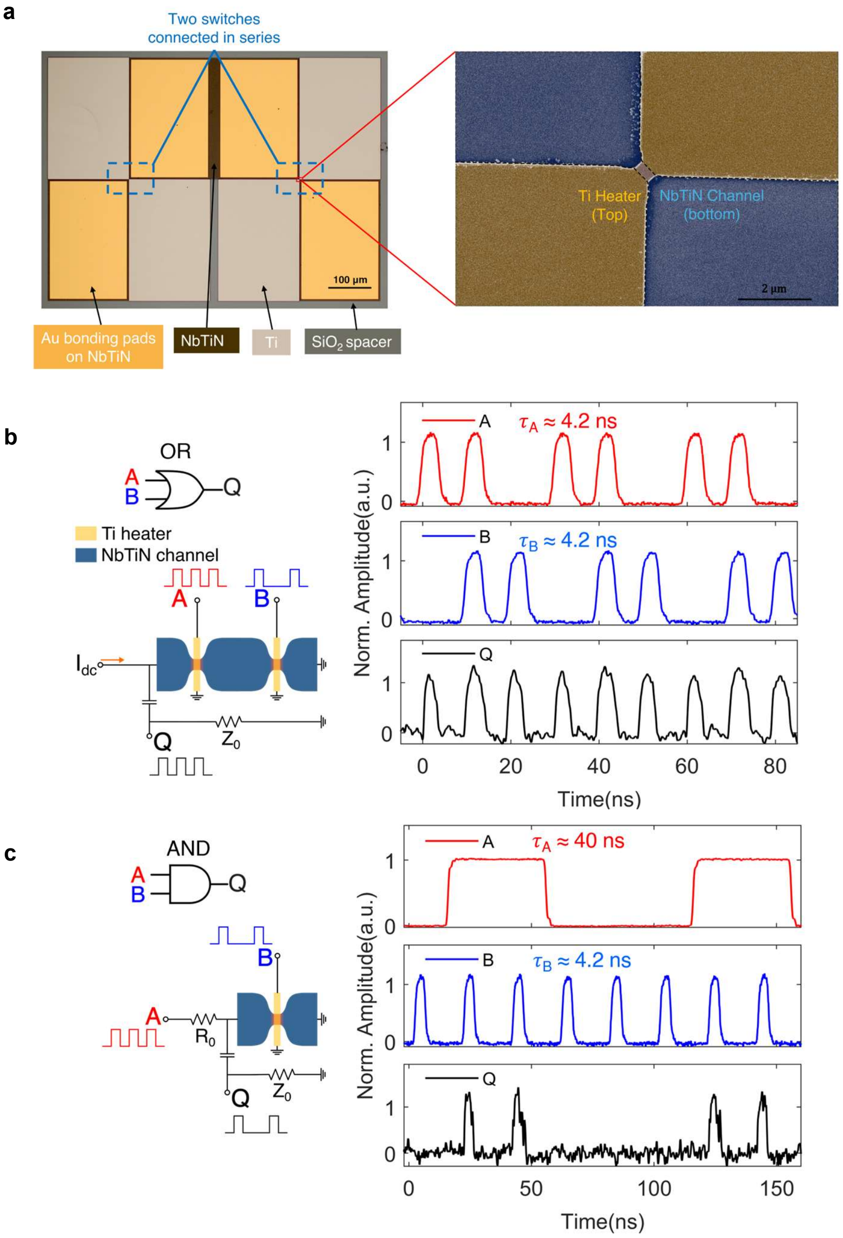

Figure S9. Implementation of the OR and AND gate. **a,** Microscope (left) and SEM (right) image of two superconducting thermal switches whose NbTiN channels are connected in series on the chip. **b-c,** Schematics (left) and rescaled input/output waveforms (right) of the OR (b) and AND (c) gates built using superconducting thermal switches.

The schematics and experimental results of the OR and AND gates are depicted in Figure S9. To build a OR gate, two superconducting thermal switches were connected in series as shown in Figure S9a, and biased with a current source. Here the input impedance of the amplifier $Z_0 = 50\ \Omega$ in parallel with the superconducting switches acted as the load resistor in the experiment, which was coupled through a capacitor. The experimental result in Figure S9b matches the truth table of a OR gate: the output voltage level is LOW when both of the NbTiN

channels are superconducting, and becomes HIGH when the superconductivity is broken in either channel because of the joule heating from the heater. Since the normal resistance of the NbTiN channel far exceeds the load resistor, the difference between two HIGH input signals and only one HIGH input signal is not substantial, which is analogous to the observation in the NOR gate. In Figure S9b, the energy dissipation per input pulse on the two heaters was 1.61 fJ and 1.46 fJ. Calculated from the output amplitude and the bias current, the energy consumption in the channel and the load resistor was around 164.9 aJ per operation. Therefore, the whole OR gate required around 3.23 fJ energy dissipation for one operation with both HIGH inputs.

The AND gate is shown in Figure S9c. Here, the two inputs, A and B, activate the NbTiN channel and the heater of the superconducting thermal switch, respectively. A small resistor R_0= 22 Ω was added between the input waveform generator and the NbTiN channel to avoid a short circuit. The load resistor Z_0 was placed in parallel with the NbTiN channel via a capacitor. Only when both input levels are HIGH can we obtain a HIGH voltage level at the output, which is consistent with the experimental results in Figure S9c. Assuming both of the inputs were HIGH for the pulse duration $\tau_\mathrm{B} = \tau_\mathrm{A} \approx 4.2$ ns, the switching energy dissipated in the heater was approximately $\tau_\mathrm{B} {V_\mathrm{B}}^2/R_\mathrm{h}$ = 4.2 ns × (6.4 mV)^2/194 Ω ≈ 0.887 fJ , and the energy consumption in the channel and the biasing circuit was around $\tau_\mathrm{A} {V_\mathrm{A}}^2/(R_0 + Z_0)$ = 4.2 ns × (0.28 mV)^2/(22 Ω+50 Ω) ≈ 0.00457 fJ. Thus the energy consumption of the whole gate for one output pulse was around 0.892 fJ.

8. NAND gate implementation

One approach to construct a NAND gate is presented in Figure 3c in the main text, where both the superconducting channel and the heater element carry the input signals. Another feasible method to accomplish a NAND gate is to connect two superconducting switches in parallel, as depicted in Figure S10a. Biasing the two superconducting switches separately is advantageous to ensure a stable switching threshold, as it avoids the current redistribution between two superconducting channels. However, it can be observed from the experimental result in Figure S10b that a gate threshold must be determined so that the low level is only reached when both switches are non-superconducting. Compared with the method shown in Figure 3c, this approach is more complex since it requires more delicate designs of the device geometries and the electrical parameters in multi-stage logic circuits.

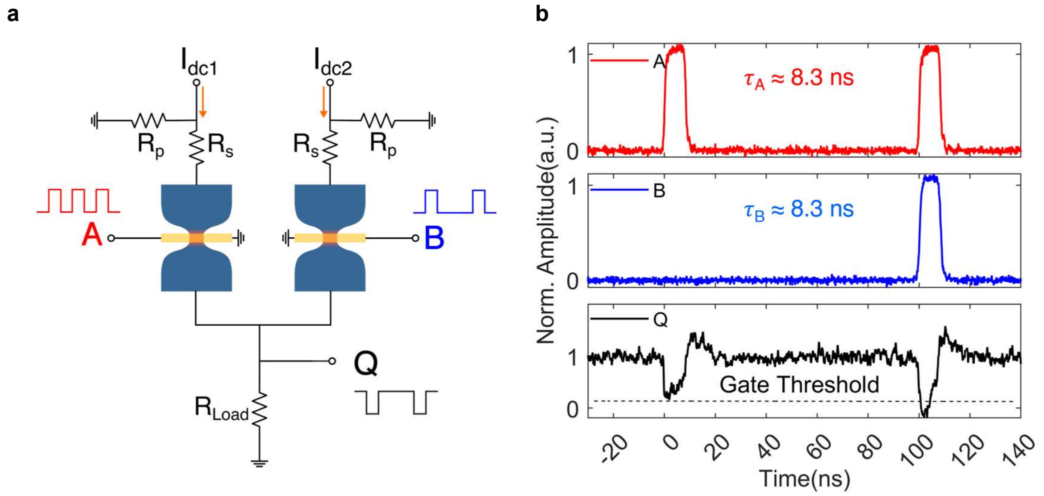

Figure S10. A NAND logic gate composed of two parallel superconducting switches. **a,** Schematic of the circuit. **b,** Rescaled experimental traces of the input and output signals.

9. Memory cell implementations

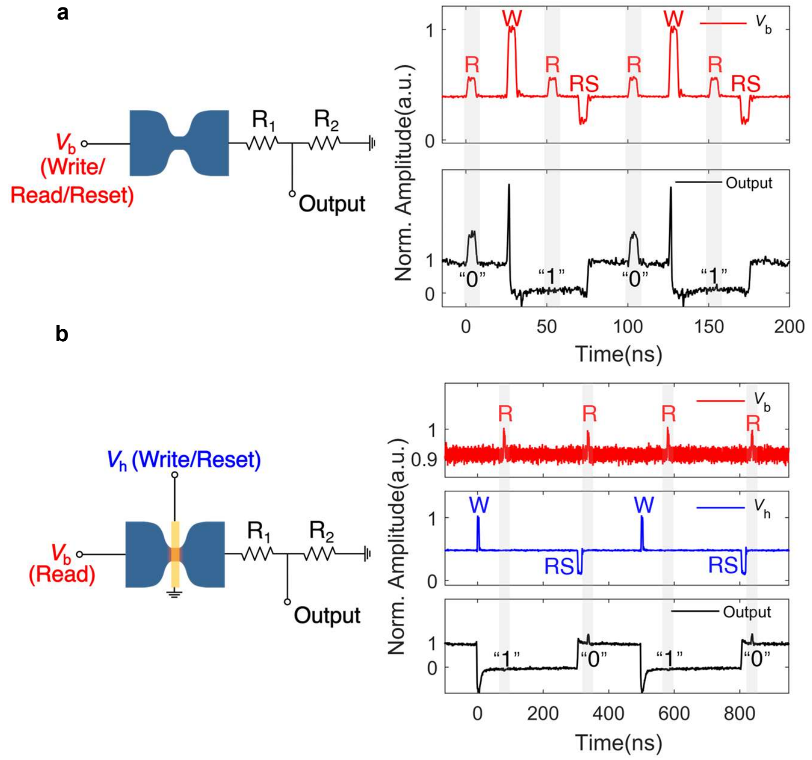

Figure S11. Alternative ways to build a memory cell. Figures on the left depicts the circuit diagrams and figures on the right show the experimental results. **a,** Write (W), Read (R) and Reset (RS) of the memory through the superconducting channel by adjusting the pulse amplitude. **b,** Write (W) and Reset (RS) of the memory via the heater, and Read (R) through the superconducting channel. The output waveform is averaged over 10 measurements for better SNR.

One way to construct a memory cell from superconducting thermal switches is shown in Figure 5, where Write and Reset is done via the heater. There are two other approaches to operate the memory cell, as shown in 10. One approach is to write, read, and reset the memory only through the superconducting channel (Figure S11a). The spike at the writing operation is a consequence of the appearance of the writing pulse and the time delay to switch the channel to its resistive state. The power consumption in the superconducting state dominates in the device, which is around 47 nW. One read operation in superconducting state consumes 572 aJ and one write operation ~1 fJ.

In the other approach demonstrated in Figure S11b, the memory is written and reset via the heater while reading through the superconducting channel. The total power consumption is approximately 94 nW in state "1" and 112 nW in state "0". A large part of these power consumptions is the static power consumption resulting from the constant bias of the heater. The pulse energies to write and read the device are around 2.82 fJ and 130 aJ, respectively.

10. Retention time measurement of the memory cell

In order to monitor the state of the memory cell, we applied Read operations at a frequency of 1 MHz and measured the output amplitudes. Since a pulse can be detected at the output only when the memory is in state "0", the amplitude of the output signal is greater when reading the state "0" than reading the state "1". The measurement taken for over 29 hours is shown in Figure S12, which indicates that both states of our memory cell could be retained for over 10^5 s.

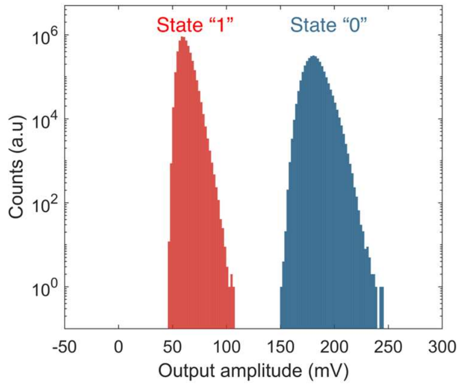

Figure S12. The measurement of the output amplitude of reading the state "0" and the state "1" for over 29 hours.

11. Comparison with literature values

As a mature platform in room temperature, CMOS technology has achieved a switching energy < 1 fJ[5], an operation frequency > 5 GHz[6], and a bit error rate < 10^{-12} with proper transistor sizes[7]. However, due to the concern of the heat load and the complexity in the integration with different semiconductors and superconductors, there remains an increasing demand to develop superconducting electronics. In Table S1 we compare the performance of our superconducting thermal logic gates or memory devices with other superconducting devices reported in the literatures, which all present their own strengths and weaknesses.

Table S1. Comparison with literature values

Technology	Ref.	Material	T (K)	Type of elements	Power consumption per device	Energy consumption per operation	Speed	Size	BER
Superconducting thermal switches	**This work**	NbTiN	3	Logic<br>Memory (non-destructive readout)	0.1-2.25 μW	0.1 to 20 fJ	100MHz	~10^{-1} μm^2	5×10^{-9}<br>8×10^{-7}
Superconducting nanowire sensors	Chen, et al. (2024)[8]	Nb	6.5	Memlogic (non-destructive readout)	~600 μW	/	~kHz	10^4 μm^2	/
Superconducting loops with nanocryotrons (nTrons)	Buzzi, et al. (2023)[9]	NbN	4.2	Memlogic (destructive readout)	/	1 fJ	50 MHz	~25 μm^2 [a]	/
Superconducting loops with hTrons	Butters, et al. (2021)[10]	NbN	4.2	Memory (destructive readout)	/	/	Tens of MHz	~10 μm^2 [a]	10^{-17} [b]
Nanowire cryotrons (hTrons and yTrons)	Zhao, et al. (2018)[11]	NbN	4.2	Memory (destructive or non-destructive readout)	/	10 fJ	Tens of MHz	21 μm^2	10^{-10}-10^{-7}
Nanocryotrons (nTrons)	McCaughan & Berggren (2014)[12]	NbN	4.2	Logic	/	10 aJ[c]	1.2 Hz	~10^{-1} μm^2	1.75×10^{-3}
Superconducting memristors	Alam, et al. (2021)[13]	Nb/AlO_x/Nb and Al/AlO_x/Al	/	Memory	/	0.1-1 aJ	/	1-10 μm^2	/
Coupled Superconductor-Insulator-Superconductor Josephson junction arrays	Nair, et al. (2019)[14]	Nb/AlO_x/Nb	/	Memory	/	0.011-0.23 aJ	>10 GHz	/	/
Rapid single-flux-quantum (RSFQ) technology	Chen, et al. (1999)[15]	Nb/AlO_x/Nb	4.2K	Logic	/	/	770 GHz	0.1 μm^2/junction	/
	Filippov, et al. (2024)[16]	Nb/AlO_x/Nb	4.2K	Logic	/	/	75 GHz	/	10^{-14} [d]

[a] Estimated from the SEM image of the device

[b] Estimated from 2000 write/read cycles

[c] Estimated by $E \sim LI^2$, where L is around 1 nH and I is around 100 μA.

[d] Estimated from the probability function of switching, which depends on the measurements of the grey zone threshold of the balanced comparator

12. Supplementary figures without amplitude normalization

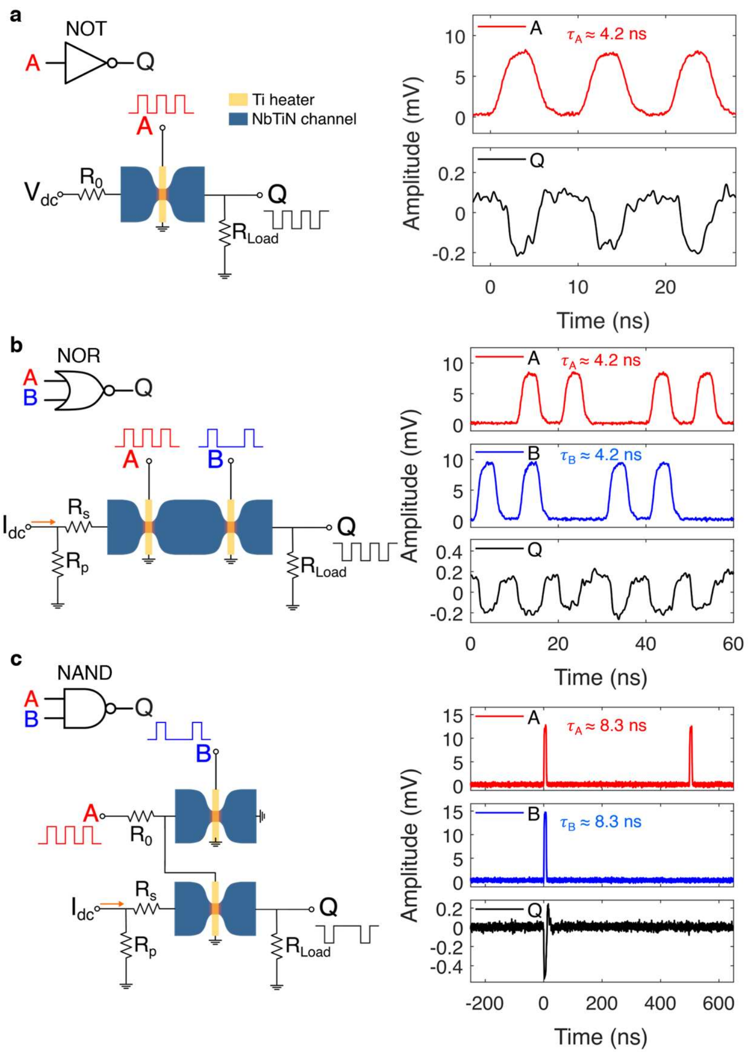

Figure S12. Implementation of fundamental logic gates with the amplitudes unnormalized. Attenuations or amplifications have been considered and corrected in the figure. **a-c,** Implementation of fundamental logic gates NOT (a), NOR (b), and NAND (c) using superconducting thermal switches. The resistive elements shown in the figures are $R_0 = 22\ \Omega$, $R_{\mathrm{Load}} = 50\ \Omega$, $R_{\mathrm{p}} = 100\ \Omega$, and $R_{\mathrm{s}} = 50\ \Omega$.

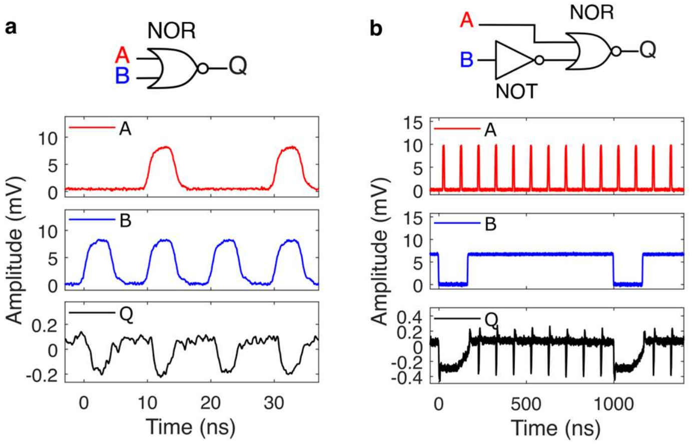

Figure S13. Implementation of the logic operations with the amplitudes unnormalized. Note: signal attenuations or amplifications have been considered and corrected in the figure. **a and b,** Unnormalized plots for the logic operations demonstrated in Figure 4a (a NOR gate) and Figure 4b (a NOT gate and a NOR gate).

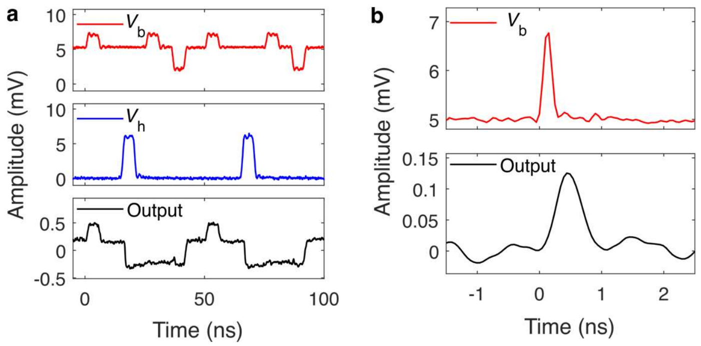

Figure S14. Implementation of a memory cell with the amplitudes unnormalized. Note: signal attenuations or amplifications have been considered and corrected in the figure. **a and b,** Unnormalized plots for the memory operations demonstrated in Figure 5b (Read/Reset with the superconducting channel and Write with the heater) and Figure 5c (Read operation with sub-nanosecond pulses).

References

1. Gourgues, R. *et al.* Superconducting nanowire single photon detectors operating at temperature from 4 to 7 K. *Opt. Express* **27**, 24601-24609 (2019). https://doi.org:10.1364/OE.27.024601
2. Zichi, J. *et al.* Optimizing the stoichiometry of ultrathin NbTiN films for high-performance superconducting nanowire single-photon detectors. *Opt. Express* **27**, 26579-26587 (2019). https://doi.org:10.1364/OE.27.026579
3. Shakeripour, H., Petrovic, C. & Taillefer, L. Heat transport as a probe of superconducting gap structure. *New Journal of Physics* **11** (2009). https://doi.org:10.1088/1367-2630/11/5/055065
4. McCaughan, A. N. *et al.* A superconducting thermal switch with ultrahigh impedance for interfacing superconductors to semiconductors. *Nature electronics* **2** (2019). https://doi.org:10.1038/s41928-019-0300-8
5. Datta, S., Chakraborty, W. & Radosavljevic, M. Toward attojoule switching energy in logic transistors. *Science* **378**, 733-740 (2022). https://doi.org:10.1126/science.ade7656
6. Lee, T. H. & Wong, S. S. CMOS RF integrated circuits at 5 GHz and beyond. *Proceedings of the IEEE* **88**, 1560-1571 (2000). https://doi.org:10.1109/5.888995
7. Ibrahim, W., Beiu, V. & Beg, A. Optimum Reliability Sizing for Complementary Metal Oxide Semiconductor Gates. *IEEE Transactions on Reliability* **61**, 675-686 (2012). https://doi.org:10.1109/TR.2012.2206249
8. Chen, B. *et al.* Reconfigurable memlogic long wave infrared sensing with superconductors. *Light: Science & Applications* **13**, 97 (2024). https://doi.org:10.1038/s41377-024-01424-2
9. Buzzi, A. *et al.* A nanocryotron memory and logic family. *Applied Physics Letters* **122**, 142601 (2023). https://doi.org:10.1063/5.0144686
10. Butters, B. A. *et al.* A scalable superconducting nanowire memory cell and preliminary array test. *Superconductor Science and Technology* **34**, 035003 (2021). https://doi.org:10.1088/1361-6668/abd14e
11. Zhao, Q.-Y. *et al.* A compact superconducting nanowire memory element operated by nanowire cryotrons. *Superconductor Science and Technology* **31**, 035009 (2018). https://doi.org:10.1088/1361-6668/aaa820
12. McCaughan, A. N. & Berggren, K. K. A Superconducting-Nanowire Three-Terminal Electrothermal Device. *Nano Letters* **14**, 5748-5753 (2014). https://doi.org:10.1021/nl502629x
13. Alam, S., Hossain, M. S. & Aziz, A. A cryogenic memory array based on superconducting memristors. *Applied Physics Letters* **119**, 082602 (2021). https://doi.org:10.1063/5.0060716
14. Nair, N., Jafari-Salim, A., D'Addario, A., Imam, N. & Braiman, Y. Experimental demonstration of a Josephson cryogenic memory cell based on coupled Josephson junction arrays. *Superconductor Science and Technology* **32**, 115012 (2019). https://doi.org:10.1088/1361-6668/ab416a
15. Chen, W., Rylyakov, A. V., Patel, V., Lukens, J. E. & Likharev, K. K. Rapid single flux quantum T-flip flop operating up to 770 GHz. *IEEE Transactions on Applied Superconductivity* **9**, 3212-3215 (1999). https://doi.org:10.1109/77.783712
16. Filippov, T. V., Sahu, A., Kirichenko, D. E., Çelik, M. E. & Gupta, D. Experimental Evaluation of Josephson Balanced Comparators Toward 100 GHz RSFQ Circuits. *IEEE Transactions on Applied Superconductivity* **34**, 1-5 (2024). https://doi.org:10.1109/TASC.2024.3351626
